# Source Apportionment of Coarse Particulate Matter (PM_10_) in Yangon, Myanmar

**DOI:** 10.3390/ijerph17114145

**Published:** 2020-06-10

**Authors:** Piyaporn Sricharoenvech, Alexandra Lai, Tin Nwe Oo, Min M. Oo, James J. Schauer, Kyi Lwin Oo, Kay Khine Aye

**Affiliations:** 1 Environmental Chemistry and Technology program, School of Engineering, University of Wisconsin-Madison, Madison, WI 53706, USA; sricharoenve@wisc.edu (P.S.); alexandra.lai@weizmann.ac.il (A.L.); 2 Department of Medicine, School of Medicine and Public Health, University of Wisconsin-Madison, WI 53705, USA; too@wisc.edu; 3 Space Science and Engineering Center, University of Wisconsin-Madison, Madison, WI 53706, USA; min.oo@ssec.wisc.edu; 4 Wisconsin State Laboratory of Hygiene, Madison, WI 53706, USA; 5 Occupational and Environmental Health Division, Department of Public Health, Ministry of Health and Sports, Nay Pyi Taw, Myanmar; drkyiser@gmail.com (K.L.O.); kaykhineaye276@gmail.com (K.K.A.)

**Keywords:** PM_10_, coarse particulate matter, air pollution, haze, source apportionment, chemical mass balance (CMB) model

## Abstract

The Republic of the Union of Myanmar is one of many developing countries facing concerns about particulate matter (PM). Previously, a preliminary study of PM_2.5_ in 2018 suggested that the main source of PM in Yangon, the former capital, was vehicle emissions. However, this suggestion was not supported by any chemical composition data. In this study, to fill that gap, we quantitatively determined source contributions to coarse particulate matter (PM_10_) in Yangon, Myanmar. PM_10_ samples were collected in Yangon from May 2017 to April 2018 and chemically analyzed to determine composition. Chemical composition data for these samples were then used in the Chemical Mass Balance (CMB) model to identify the major sources of particulate matter in this area. The results indicate that PM_10_ composition varies seasonally according to both meteorological factors (e.g., precipitation and temperature) and human activities (e.g., firewood and yard waste burning). The major sources of PM in Yangon annually were dust, secondary inorganic aerosols (SIA), and secondary organic aerosols (SOA), while contributions from biomass burning were more important during the winter months.

## 1. Introduction

The Republic of the Union of Myanmar (i.e., Burma), located in Southeast Asia, is one of many developing countries facing increasing concerns regarding particulate matter (PM) concentrations due to its rapid economic development and urbanization. A long-term trend study by Shi et al. [1] reported that, from 1999 to 2014, PM_2.5_ (i.e., particulate matter smaller than 2.5 microns in diameter) concentrations in Myanmar significantly increased each year at a rate of 0.25 µg/m^3^/year. However, only a few studies of particulate matter in Myanmar have been conducted and limited information about the sources of PM in urban regions of Myanmar has been reported. A five-day preliminary study by Yi et al. [2] monitored the concentrations of PM_2.5_ and attempted to pinpoint the PM_2.5_ hotspots at different times of the day in seven townships in Yangon, the former capital of Myanmar. The results show that the concentrations reached the highest points in the mornings and the authors speculated that the main sources of PM_2.5_ in these townships were motor vehicle emissions. Unfortunately, the study did not include a chemical analysis or quantitative source apportionment of PM. Furthermore, currently, Myanmar does not have country-specific air quality standards, and the best reference point for this study is the WHO’s air quality guidelines for PM_10_, which is set at 20 µg/m^3^. Therefore, there is still a need to better quantify the sources of PM in Yangon to provide guidance for policymakers and researchers to develop plans to mitigate PM pollution in Yangon.

Source apportionment models are common applications that allow users to assess the sources of pollution and their contributions to outdoor air pollution. The ability to classify the types of sources, whether they are natural or anthropogenic, and whether they are local or long range-transported, is useful for preparing air quality plans and control policies [3]. The Chemical Mass Balance (CMB) model is a receptor model that estimates the contributions of particulate matter sources to the atmospheric samples. Each source profile is assigned fingerprints that indicate the source where they originated from. By using the CMB model, the users are provided with source contribution estimates and the uncertainties that can help to prioritize major sources and plan suitable control strategies.

The main goal of this work is to fill in gaps from the previous findings of PM in Yangon, Myanmar—the identity and source of chemical compositions. To understand the chemical composition, PM_10_ samples were collected every six days from May 2017 to April 2018 using a PM_10_ sampler deployed in Yangon and analyzed for chemical composition. The Chemical Mass Balance (CMB) model was then applied to identify possible sources of PM. The results show promising insights that could be helpful in managing particulate matter-related air quality in Yangon, Myanmar, in the future.

## 2. Materials and Methods

### 2.1. Study Site and Sampling Methods

Coarse particulate matter (PM_10_) was collected in Yangon, the former capital located in the southern part of the Republic of the Union of Myanmar. Yangon was the administrative capital until 2006 and as home to approximately five million people, Yangon is considered to be the largest city and the most important commercial center in the country. According to the Myanmar Climate Report [4], the climate of Myanmar can be described as having three seasons: winter, summer and the rainy season. In winter, starting from around mid-November and lasting until February, a series of northeast monsoons travel across the northern part. These turbulences moving towards the Indian Ocean bring dry cool wind with some moisture from the Gulf of Bengal to the land, resulting in a small amount of precipitation in Myanmar during this season, especially in January and February. From March to mid-May, the temperature continuously and rapidly increases, causing the formation of severe local storms, which are usually accompanied by strong winds, hail, and torrential rain. In the rainy season, from around the third week of May until November, the southwest monsoon traveling across Myanmar brings heavy rain to the land, especially in the coastal areas, which include Yangon. Although there have not been many emission inventory studies conducted in Yangon, traffic was believed to be the primary source of PM in the city. For example, Yi et al. [2] considered traffic as the major source of PM_2.5_ in Yangon. Additionally, there was some construction taking place in the city during the sampling period. Hence, dust was expected to be another major source of PM_10_ in Yangon.

Samples and field blanks were collected every six days, from May 2017 to April 2018, at the Occupational and Environmental Health Division, Ministry of Health and Sports, Ahlone, Yangon, Myanmar. The sampling design has been found to be representative of monthly average concentrations from prior studies (Lough et al. [5] and Bae et al. [6]). An ambient air PM_10_ sampler (APM 460 NL, New Delhi, India), operating at a flow rate of 1 cubic meter per minute (m^3^/min) for at least 24 h per sample, was deployed on the second-floor rooftop (approximately 6 m away from the ground) of the division’s building which is located next to one of the main roads in residential and commercial areas. The flow rate was measured before and after sampling and was within 20% of the flow rate target (1 m^3^/min). Throughout this study, 8 × 10-inch quartz fiber filters (GE Whatman, USA) were used to collect the PM_10_ samples. The filters were baked at 550 °C for at least 12 h and were stored in glassine bags prior to shipping to the sampling site. After collecting the samples, the filters were folded in half, placed in glassine envelopes, and stored in a freezer until chemical analysis.

### 2.2. Chemical Analysis

The quartz sample filters and field blanks were prepared for chemical analysis at the University of Wisconsin-Madison and the Wisconsin State Laboratory of Hygiene (WSLH). The procedures for elemental carbon (EC), organic carbon (OC), water-soluble organic carbon (WSOC), water-soluble ions (e.g., sulfate, nitrate, and ammonium), trace elements, and organic molecular marker measurements are explained by Secrest et al. [7], and Skiles et al. [8].

The quartz filters were weighed pre- and post-analysis using a microbalance (MT 5, Mettler-Toledo Inc., Hightstown, NJ) in a climate-controlled environment (23 °C and 40% relative humidity) for mass concentrations. Six 1 cm^2^ punches were then taken from each filter for the chemical analyses. The first punch was submitted to the trace element clean laboratory at WSLH to measure EC and OC concentrations using a thermos-optical EC/OC analyzer (UW-TOT, Sunset Labs, Tigard, OR) by following the modified NIOSH 5040 protocol [9].

Another two punches were used to measure WSOC and water-soluble ion (Na^+^, K^+^, NH_4_^+^, Ca^2+^, Cl^-^, NO_3_^-^, and SO_4_^2-^) concentrations at the Water Science and Engineering Laboratory (WSEL) at the University of Wisconsin-Madison. Each punch was extracted in 15 mL of Milli-Q water, shaken for 6 h, filtered through pre-cleaned syringes with 0.45-μm polypropylene syringe filters (Whatman Paradisc, Thermo Fisher Scientific, USA), and collected in acid-washed glass vials. The collected solutions were then analyzed for WSOC concentration using a total organic carbon (TOC) analyzer (Siever M9, GE Analytical Instruments, Boulder, CO). Then, 2 mL of the remaining solution of each sample was analyzed for water-soluble ions using ion chromatography (Dionex ICS 2100 for anions and Dionex 1100 for cations; Thermo Fisher Scientific, USA).

The last three punches were submitted to the trace element clean laboratory at WSLH and analyzed for the concentrations of 40 trace elements (Al, Si, K, Sc, Ti, V, Cr, Mn, Fe, Co, Ni, Cu, Zn, Se, Rb, Sr, Y, Mo, Ag, Cd, Sn, Sb, Cs, Ba, La, Ce, Pr, Nd, Sm, Eu, Dy, Ho, Yb, Lu, W, Pt, Tl, Pb, Th, U). The punches were microwave digested in an acid digestion solution (1 mL of 6M HNO_3_, 0.25 mL of 12M HCl, and 0.1 mL of HF), diluted with Milli-Q water, and measured for the trace element concentrations in each sample using inductively coupled plasma mass spectrometry (ICPMS; Thermo-Finnigan Element 2; Thermo Fisher Scientific, USA).

For the analysis of organic molecular markers, portions of the remaining filters were grouped by month. The monthly composite samples were spiked with either 100 or 250 μL of a mixture of internal standards corresponding to the compounds to be analyzed, depending on filter loading, and extracted by Soxhlet in a 50:50 methylene chloride/acetone mixture [10]. The final volume of each sample was reduced to 100 or 250 μL (the same volume as the internal standard added to that sample) and divided into 2 aliquots. The first aliquot was silylated to measure levoglucosan and secondary organic carbon (SOC) tracer concentrations. The second aliquot was methylated to measure other organic molecular marker concentrations using gas chromatography-mass spectrometry (GCMS) (GC: 6890, MS: 5973, column: DB-5 Capillary column; Agilent Technologies, USA).

Quality assurance and quality control processes were performed along with the chemical analyses to ensure the precision and accuracy of the results. The number of field blanks and analysis blanks used in this study was equal to 10% of the number of total samples. The coefficient of variation (CV) values of WSOC, water-soluble ions, and trace element measurements were below 10% for most of the samples with duplicates, indicating that the measurements had high precision. Likewise, high sample concentration to field blank concentration ratios (varied from 2.8 to 192.4) of almost all trace element measurements also indicated low contamination due to sampling.

### 2.3. Chemical Component Classification

The detected chemical components were divided into 8 categories: elemental carbon (EC), organic matter (OM), dust, sulfate, nitrate, other water-soluble ions (i.e., chloride, phosphate, ammonium, and water-soluble potassium ion), trace elements, and other species. Organic matter (OM) was estimated by multiplying a factor of 1.8 [11]. The concentration of dust was estimated from the major crustal elements, including SiO_2_, Al_2_O_3_, K_2_O, TiO_2_, CaO, MgO, MnO_2_, using the following expression [12,13]:

Dust = 2.14[Si] + 1.89[Al] + 1.40[Ca] + 1.66[Mg] + 1.67[Ti] + 1.43[Fe] + 1.58[Mn] + 1.20[K](1)

The concentration of silicon (Si) concentration was not directly measured by ICPMS; therefore, it was estimated using a silicon to aluminum ratio of 4.6 [13]. The portion of “other” species assigned for unidentified or uncategorized species was calculated by subtracting the sum of the concentrations of every species from the mass concentrations.

### 2.4. Statistical Analysis

Principal component analysis (PCA) was conducted using the “psych” package (version 1.5.8) performed in R (version 3.5.1). The dataset used in the PCA contained any of the species measured for individual samples (inorganic trace elements measured by ICPMS, soluble inorganic ions measured by ion chromatography (IC), EC, water-insoluble organic carbon (WIOC), and WSOC) where greater than 80% of samples were statistically non-zero after blank subtraction [14]. Only factors with eigenvalues greater than one were considered. The factors from PCA were then used for identifying the potential sources of PM_10_ in Yangon, Myanmar.

### 2.5. Source Apportionment

Chemical mass balance (CMB) is a receptor model. The modeling was used to identify the sources of PM_10_ collected in Yangon, Myanmar, from May 2017 to April 2018, using the CMB8.2 program developed by the US EPA. The source profiles used in this study represented vegetative detritus, vehicle tailpipe emissions, biomass burning, and coal combustion [15,16,17,18]. Vegetative detritus is dead particulate organic material from parts of plants. The vegetative detritus source profile used in this study was adopted from a study on particulate matter emitted from the abrasion of leaves by Rogge et al. [13]. Vehicle source profiles were adopted from Lough et al. [16], where organic molecular markers were measured in emissions from diesel-powered, gasoline-powered, and smoking (non-catalyzed) vehicles. The source profile of biomass burning was derived from the combustion of jackfruit branches, as reported by Sheesley et al. [17]. A study by Sheesley et al. [19] showed that CMB results are not sensitive to biomass profiles. Thus, the jackfruit branch combustion profile can be used as a representative of regional biomass material (e.g., gardening waste) burning profiles in Yangon. Lastly, the source profiles of coal combustion emissions were adopted from Chinese residential coal combustion data reported by Zhang et al. [18].

## 3. Results

### 3.1. Bulk Chemical Composition

**Figure 1:**
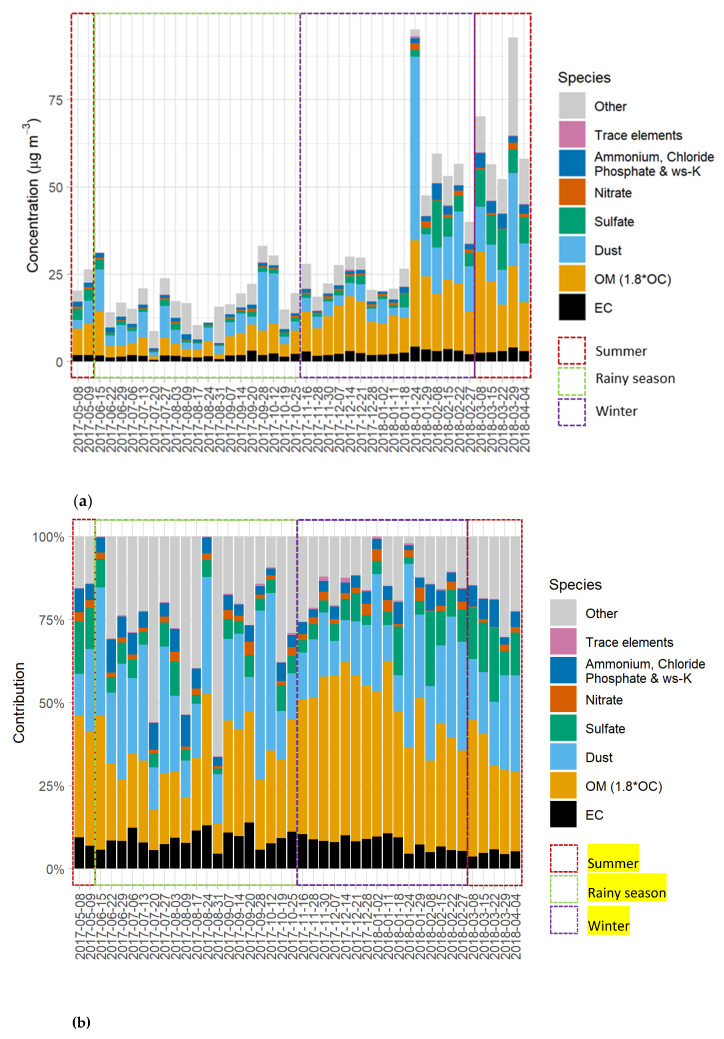
(**a**) Mass reconstructed concentrations of daily coarse particulate matter (PM_10_) in Yangon, Myanmar. (**b**) Chemical component contributions in daily PM_10_ in Yangon, Myanmar.

**Figure 2:**
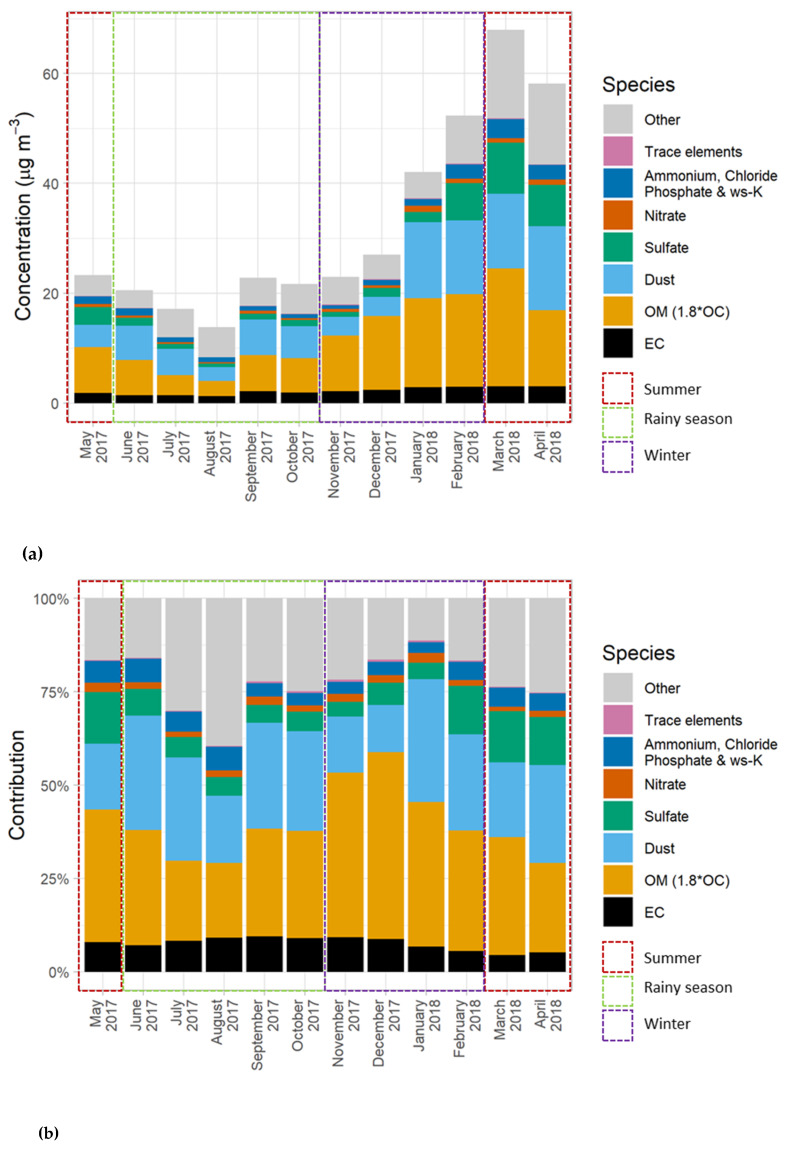
(**a**). Mass reconstructed concentrations of monthly PM_10_ in Yangon, Myanmar. (**b**) Chemical component contributions in monthly PM_10_ in Yangon, Myanmar.

The highest PM_10_ mass concentration was observed in summer (49.8 ± 7.8 µg/m^3^), followed by the winter (36.1 ± 3.4 µg/m^3^) and the rainy season (19.3 ± 0.7 µg/m^3^). As seen in Figure 1a and Figure 2a, higher PM_10_ mass concentrations from the second week of January (late winter) to the first week of April (summer) compared to those of other periods indicated a seasonal pattern. When considering the contributions of each species, OM and dust shared the majority of identified chemical components (19.4–50.0% and 14.0–33.6%, respectively) in PM_10_ as shown in Figure 1b and Figure 2b. These seasonal trends may have resulted from turbulence and strong winds during winter and summer, which could resuspend dust and organic matter in the atmosphere, while rain removes particles from the air during the rainy season. Dust was believed to originate primarily from ongoing construction during the sampling period. Meanwhile, OM was believed to mainly consist of bioaerosols, which will be further discussed in Section 3.2.2. It is worthwhile mentioning that the monthly average concentrations presented in Figure 2a,b are a useful piece of material that informs practical seasonal/monthly control policies, and prevents policymakers from misinterpreting the changes affected by some extreme events that occurred during the study period. It is also important to note that this study did not quite cover one complete year, as sampling collection stopped at the first week of April. However, this data set still includes all twelve months, sufficient to indicate trends of Yangon PM_10_ concentrations throughout the year.

**Figure 3:**
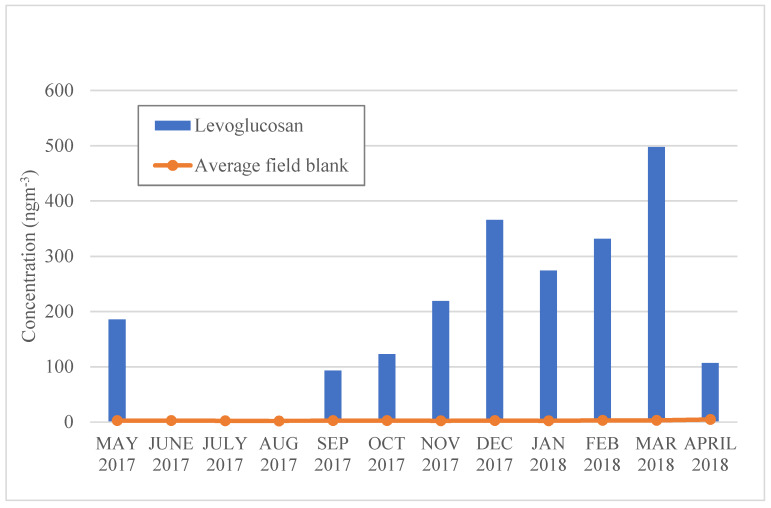
Monthly levoglucosan concentrations in Yangon, Myanmar.

Levoglucosan concentrations were highest in the winter and spring months (Figure 3). Levoglucosan is a highly specific molecular marker for biomass burning emissions used in many source apportionment studies [20,21,22,23]. It originates from cellulose pyrolysis and is stable when emitted in a large amount to the atmosphere [20]. Key sources of levoglucosan include wood fuel combustion during winter, agricultural waste combustion, and forest fires [24]. The highest levels of levoglucosan were reached in March 2018, followed by December 2017 and February 2018 (497.5, 365.5 and 331.3 ng/m^3^, respectively). These high levels might indicate ambient PM_10_ contributions from agricultural waste management based on these seasonal trends. Burning agricultural residues for land clearing is a common practice in Asian countries, such as India, Pakistan, Myanmar, Thailand, Laos, Vietnam, and Cambodia [25,26]. According to a recent Global Information and Early Warning System on Food and Agriculture (GIEWS) Brief Report [27], the harvesting periods of some major crops (e.g., maize and rice) in Myanmar are usually in February and March. The peak in December 2017 could also be attributed to agricultural waste management, as the harvesting period for maize usually takes place from November to January. Even though Yangon is an urban environment with few agricultural fields [28], the levels of levoglucosan concentrations in PM would be slightly increased after the land clearing. Likewise, Yangon has been known to have problems with yard waste management that could possibly contribute levoglucosan to the ambient air. According to an article in Myanmar Times [29], city garbage trucks do not collect yard waste, resulting in people being pushed to solve this problem by burning yard waste. Additionally, wood fuel burning in households could also be a plausible explanation for the increases in levoglucosan levels in winter. As stated by the Myanmar Energy Consumption Surveys Report [30], more than half of households in Yangon’s townships use firewood as an energy source for cooking and heating. Therefore, the level of levoglucosan concentration might be the result of households using firewood. In contrast to winter, concentrations of levoglucosan were below the detection limit from June to August. These below-detection levels could be attributed to scavenging by rain, as this is during the rainy season, and less firewood or agricultural waste burning during these times.

**Figure 4:**
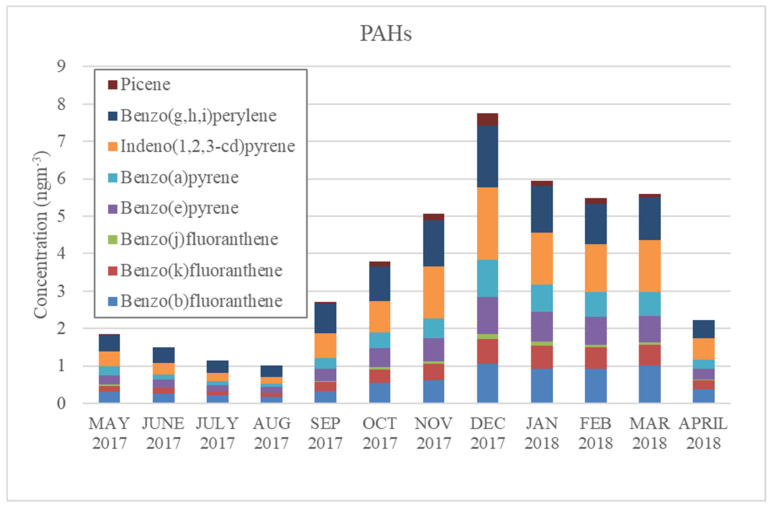
Monthly concentrations of polycyclic aromatic hydrocarbons (PAHs) in Yangon, Myanmar.

**Figure 5:**
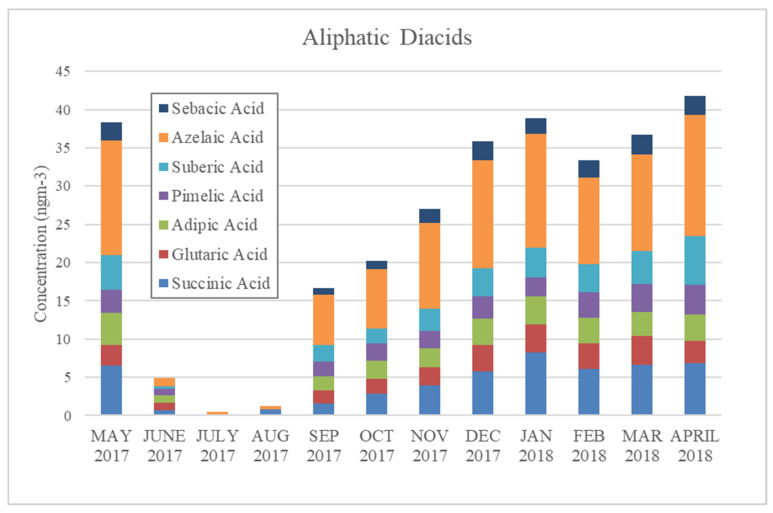
Monthly concentrations of aliphatic diacids in Yangon, Myanmar.

Polycyclic aromatic hydrocarbon (PAH) and aliphatic diacid concentrations in Yangon were also lower during the rainy season. PAHs are hydrocarbons containing multiple aromatic rings, and numerous PAHs are mutagenic and/or carcinogenic [31,32]. PAHs are byproducts of incomplete combustion and are emitted from both natural sources and anthropogenic sources, including fuel oil, gasoline, coal, firewood, and tobacco smoking [31,33]. PAH levels in Yangon increased in September and dropped dramatically in April, when precipitation started frequently occurring, as seen in Figure 4. Levoglucosan concentrations also peaked during this period, suggesting that biomass burning could be a source of these PAHs. On the other hand, aliphatic diacids, markers for secondary organic aerosols [5], showed different trends: concentrations increased in the winter and summer, and dropped only in the rainy season (Figure 5). These trends in PAHs and aliphatic diacids might have resulted from the frequent activity of rain scavengers occurring during the rainy season that removed these organic compounds from the ambient air.

**Figure 6:**
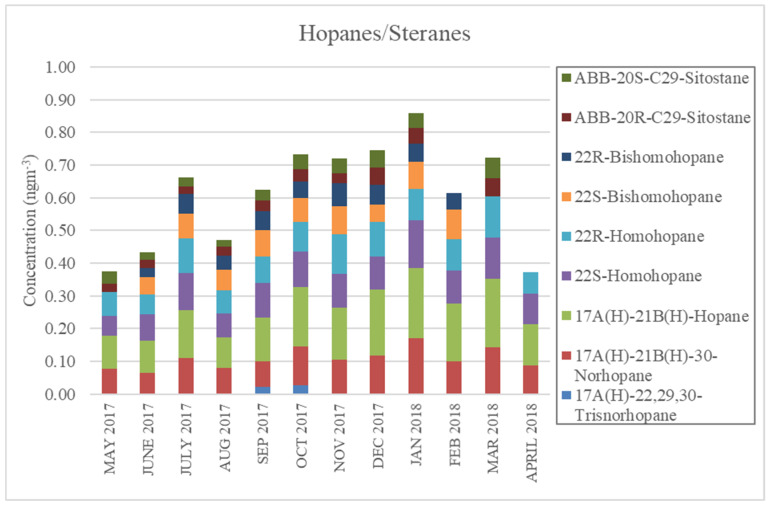
Monthly concentrations of hopanes and steranes in Yangon, Myanmar.

Hopanes and steranes, however, did not seem to have clear seasonal trends. Hopanes and steranes are groups of organic compounds commonly used as tracers of motor vehicle exhausts in urban areas due to their specificity to the lubricant oil used in diesel and gasoline engines [34,35,36,37,38]. The first three highest concentrations of hopanes and steranes occurred in all three seasons, as seen in Figure 6. Unlike other groups of organic tracers that had lower concentrations in the rainy season, these peaks suggested that there were no meteorological or seasonal factors majorly impacting hopane and sterane concentrations.

### 3.2. Source Apportionment

Daily and monthly concentrations of chemical components were used to identify sources and quantify source contributions of PM_10_ in Yangon, Myanmar during the sampling period (May 2017-April 2018). Three methods were applied: principal component analysis (PCA) to identify the sources of inorganic PM, reconstructed mass of PM_10_ to observe major inorganic and organic PM mass contributors, and the chemical mass balance of organic matter (OM) to identify sources of organic carbon.

#### 3.2.1. Source Identification and Mass Reconstruction

Principle component analysis (PCA) was used to identify the possible sources of PM_10_ using the daily concentrations of inorganic species. As seen in Table 1, the inorganic PM_10_ in Yangon, Myanmar, might have originated from five sources: dust (54.1%), secondary aerosols (13.8%), metal manufacturing (8.8%), biomass burning (8.5%) and roadway emissions (5.2%).

**Figure 7:**
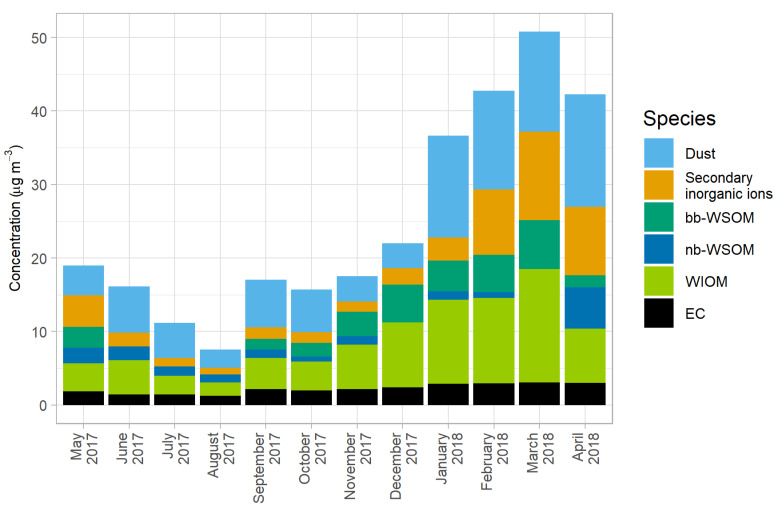
Pragmatic source apportionment of monthly PM_10_ in Yangon, Myanmar.

Carbonaceous particles and aerosol mass closure were used to preliminarily observe the major sources of inorganic and organic mass contributors of PM_10_ before running the CMB model. OM was divided into three categories: water-insoluble organic matter (WIOM), biomass burning water-soluble organic matter (bb-WSOM), and non-biomass burning water-soluble organic matter (nb-WSOM). WIOM can be calculated by multiplying WIOC (calculated by subtracting WSOC from OC) by a factor of 1.8. Dust was the major contributor to PM_10_ in almost every month, followed by WIOM and secondary aerosols (both inorganic and organic; Figure 7). When comparing each individual contributor, seasonal trends were quite clear for dust and secondary inorganic ions. The highest concentration of dust was observed in the summer, followed by the winter and the rainy season (12.2 ± 2.2, 9.5 ± 1.6 and 5.7 ± 0.4 µg/m^3^, respectively). This might be due to atmospheric turbulence and strong winds during late winter and summer.

Secondary organic aerosols (SOA) and secondary inorganic aerosols (SIA) were also major contributors to PM_10_ mass and seemed to have contrasting seasonal trends. As seen in Figure 7, SIA concentrations increased in the winter, reaching the highest point in March (12.0 µg/m^3^) in the summer, then decreasing until reaching the lowest point in August (0.9 µg/m^3^) during the rainy season. This change in SIA concentrations might be related to the seasonal variations in rainfall, as Myanmar receives the least and highest amount of precipitation in the winter and the rainy season, respectively [4]. In an ambient PM_2.5_ study in China by Fu et al. [39], the amount of precipitation was one of the factors involved in the physical removal/dilution of SIA. In other words, more precipitation in the rainy season would also affect SIA concentrations relative to other seasons in Myanmar. Furthermore, the same study [39] also stated that dust could significantly increase sulfate formation on reactive interfaces for heterogenous SO_2_ generation. Hence, higher dust concentrations in the summer and late winter would increase the concentrations of SIA compared to the rainy season. In contrast, the opposite trend was observed for SOA concentrations, as the concentrations seemed to be related to the temperature. Secondary organic aerosols (SOA) are particulate matter consisting of organic compounds (e.g., organic nitrates and sulfates) formed through atmospheric reactions. The highest SOA concentration was observed in the summer (2.6 ± 1.6 µg/m^3^ in April 2018), followed by the rainy season and the winter (1.2 ± 0.2 and 0.8 ± 0.3 µg/m^3^, respectively). This result agrees with the findings reported by Warren et al. [40] that the production of SOA is temperature dependent.

As a preliminary estimate of biomass burning contributions, WSOC from biomass burning (bb-WSOC) can be estimated by multiplying levoglucosan concentration by a biomass burning levoglucosan/OC ratio (0.0931 as reported by Sheesley et al. [19]) and a water-solubility factor of 0.71 [41]. The nb-WSOC, representing secondary organic aerosols (SOA), can then be calculated by subtracting bb-WSOC from total WSOC. Then, bb-WSOM and nb-WSOM were calculated by multiplying bb-WSOC and nb-WIOC by a factor of 2. Interestingly, there was no biomass burning-WSOM (bb-WSOM) from June to August 2017. Another plausible explanation, other than PM scavenged by rain, is that those months are in the rainy season. Fewer electric power outages occur due to consistent hydro-power supply, and therefore less biomass is used as a supplementary household energy source. Additionally, there is less biomass combustion from organic waste management in an urban area such as Yangon. Household biomass burning also supports trends in the winter (Figure 7): biomass burning contributions to PM_10_ increased with the start of winter in November, and biomass burning-WSOM reached its largest contribution in December, which usually has the lowest monthly-averaged temperature.

EC was the only species in reconstructed mass balance that did not exhibit seasonal variation during the study period. This species is typically used as a marker of diesel combustion emission [42]. In Myanmar, diesel generators in mini-grids are a major alternative source for household electricity generation [43]. Therefore, the same concentrations of EC detected throughout the sampling period could be attributable to the consistent necessity of household energy generation in addition to diesel vehicle emissions.

Prior to running the CMB model, the carbon preference index (CPI)—the ratio of odd n-alkanes to even n-alkanes—was calculated to determine whether the origin of C_24-33_ n-alkanes is biogenic detritus or anthropogenic emission (e.g., vehicles). As an example of biogenic detritus, Schmidl et al. [44] and Gonçalves et al. [45] reported odd and even n-alkanes emitted from leaves, garden, and agricultural waste burning. CPI values close to 1 indicate fossil fuel burning mostly from anthropogenic activities, while values higher than 2 indicate biogenic detritus. July and August 2017 were the only 2 months that had CPI values below 1, suggesting strong biogenic influence rather than anthropogenic sources such as vehicles, as Yi et al. [2] proposed in a preliminary PM study in Myanmar. However, there are other sources of alkanes in PM_10_ that may also explain this CPI. A study by Alves et al. [46] reported that odd and even n-alkanes contributions to PM_10_ road dust in Portugal originated from a varied range of sources, including weathered pavement materials, vehicular exhaust, lubricant oils, tire/brake debris, cooking processes, and biomass burning. Road dust composition was also affected by differences in pavement materials, traffic conditions and surroundings. Therefore, having CPI values which exceeded 1 in this study might not result only from the vehicular exhaust and biogenic emission, but also from additional sources, such as road dust and agricultural waste burning.

#### 3.2.2. CMB Source Apportionment of PM_10_ OC

**Figure 8:**
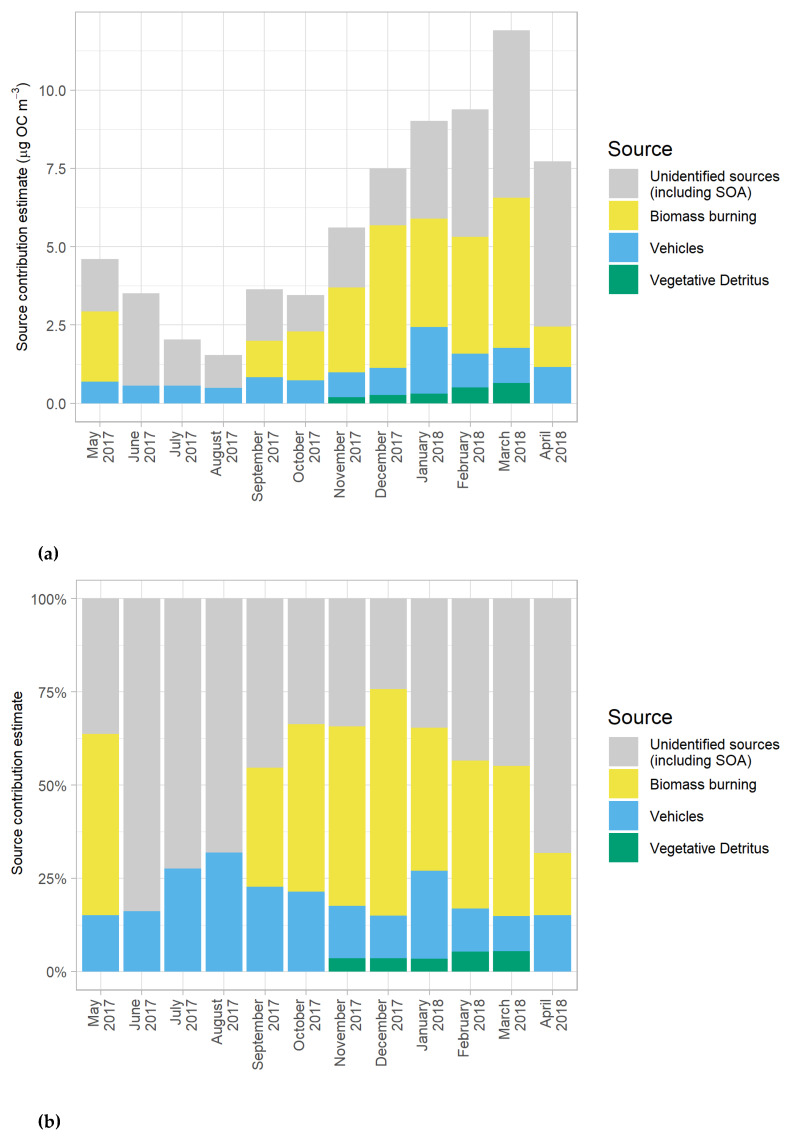
(**a**) Monthly source contributions to PM_10_ organic carbon (OC) on absolute scales estimated using the Chemical Mass Balance (CMB) model in Yangon, Myanmar; (**b**) Monthly source contribution to PM_10_ OC on percent scales estimated using CMB model in Yangon, Myanmar.

The CMB model was used to estimate monthly source contributions to OC from four major sources: vegetative detritus, vehicles, sub-bituminous residential coal combustion, and biomass burning, as seen in Figure 8a,b. A sub-bituminous coal burning profile was included in the CMB model as picene was detected in almost every monthly profile and sub-bituminous is used for energy production in Myanmar [47]. Likewise, the biomass burning source profile was included in the model as levoglucosan was detected in the monthly profiles. Diesel and smoking vehicle profiles were combined into one “vehicles” profile in this study. These profiles were developed by Lough et al. [16] using the data from varieties of California vehicles, and were considered suitable for this study because both the vehicles in Lough et al. [16] and in this study were believed to run on similar petroleum-based motor oils. Although these USA vehicle profiles may represent somewhat different automobile engine technologies from those used in Myanmar, even the most comprehensive source testing programs have large uncertainties in source profiles so that ultimately, the specificity of vehicle source profiles has limited impact on the total apportionment of the mobile sources [48]. The unidentified portion was calculated by subtracting the contribution of each source from the total source contribution. In the context of the CMB model, SOA concentrations were assumed to be included in the unidentified portion of the CMB results.

Biomass burning and vehicles were the major sources contributing to identified OC in coarse particulate matter in Yangon, Myanmar, from May 2017 to April 2018. The contribution from biomass burning started to increase in September 2017, reached the highest point of 4.6 ± 0.4 µg/m^3^ in December 2017, and decreased significantly in April 2018, when the precipitation started to frequently occur. The biomass burning source also had no contribution to OC during the rainy season (June to August 2017). As with changes in levoglucosan and bb-WSOM levels discussed previously, the change in biomass burning source contribution was related to seasonal variability in rain scavenging, agricultural waste management and firewood burning.

Vehicles, on the other hand, did not show any seasonal variation in OC contribution. Vehicle contributions did demonstrate a slight increase throughout the study period since vehicles increased their contribution to OC from 0.7 ± 0.1 µg/m^3^ in May 2017 to 1.2 ± 0.1 µg/m^3^ in April 2018. One extreme value among vehicle contributions (2.1 ± 0.9 µg/m^3^) was observed in January 2018; this was due to having both diesel and smoking vehicles as the contributors in this profile while having only the diesel vehicles in other month profiles due to collinearity. Also, it is important to note that the CMB model is unable to distinguish between the diesel exhaust of the diesel vehicles and the diesel generators used to produce electricity for Myanmar’s households. However, the diesel generators are not used on regular basis, so the major share of diesel combustion emissions would still be from diesel vehicles.

Vegetative detritus (i.e., debris of leaves and other parts of tress) also showed seasonal variability, contributing substantially only in the winter months. The contribution to OC from vegetative detritus started to increase in November 2017 until reaching the largest contribution of 0.7 ± 0.2 µg/m^3^ in March 2018. According to Davis [49], Yangon is located in a tropical dry deciduous forest zone, where leaves fall during the dry months. This implies that more coarse and fine particles from leaves of deciduous plants would have more influences on the ambient air in the winter compared to in the rainy season or the summer, when more rain can wash down the particles.

The results from the CMB model found no contribution from residential sub-bituminous coal burning. As picene, a specific fingerprint for tracing coal burning [18], was detected in almost every month, the coal combustion was expected to share some contribution to OC in each monthly profile. Additionally, residential coal combustion has a tendency to emit coal soot due to low temperature and degree of combustion. However, none of the CMB results reported any contribution from the coal combustion. This suggests that picene concentrations were very small compared to those of the tracers of other contributors, and coal combustion possibly emitted other chemical species that were categorized in different contributors. Thus, sub-bituminous coal combustion did not contribute meaningfully to PM_10_ in this study.

In contrast, the unidentified OC represented the largest contributions in 7 out of 12 months (June through September 2017 and January through April 2018). These contributions varied from 24.3% (December 2017) to 83.8% (June 2017) of PM_10_ OC, with the largest absolute contribution of 5.4 ± 2.0 µg/m^3^ in March 2018 (Figure 8a,b). OC contributions from unidentified sources also varied seasonally: the contributions started to drop in June 2017 (i.e., the beginning of the wet season), reached the smallest contribution in December 2017, and started to increase again when the temperature increased. This pattern was similar to the changes in the level of nb-WSOM representing SOA, which supports our hypothesis that SOA was the main component in the unidentified OC source in the CMB model. Looking at the beginning and end of the study period, OC contribution from the unidentified source was lower in May 2017 (1.7 ± 0.7 µg/m^3^) than in April 2018 (5.3 ± 0.7 µg/m^3^). This almost five-fold increase cannot be explained by seasonal trends, and instead might have resulted from an altered precipitation cycle and more photochemical reactions caused by warmer regional temperature. According to Slagal [50], as a result of climate change and increasing global/regional temperature, almost every part of Myanmar has been experiencing annual changes in precipitation amounts with unpredictable rainfall patterns for decades. This includes some extreme rainfall events, such as thunderstorms, that can strongly influence PM levels in the atmosphere. Another plausible explanation would be that the levels of SOAs might have been affected by dust and nitrate levels. Normally, SOAs are commonly found in a form of dicarboxylic acids generated from primary emission sources (e.g., vehicle exhausts) or produced via atmospheric photochemical processes [51]. For example, using oxalic acid (i.e., a simple dicarboxylic acid) as a case study, Wang et al. [51] proposed a three-step SOA formation process: gaseous nitric acid and/or nitrogen oxides react with calcium on dust to create a liquid phase on the particle surface, gas-phase volatile organic carbons (VOCs) partition into the liquid surface, and are subsequently oxidized to keto-carboxylic acids and/or polymerized into oligomers and eventually oxidized to oxalic acid. Simply put, nitrate and dust play important roles in SOA formation. The results from the CMB model are therefore consistent with findings from Wang et al. [50], who suggested that the contributions from unidentified OC sources (including SOA) decreased during the rainy season, when there was heavy precipitation removing atmospheric dust particles, and increased in the winter and the summer, corresponding to increases in dust and nitrate concentrations. In addition to the presence of SOA in the “unidentified” source, bioaerosols might be one of the contributors to the unidentified OC. Bioaerosols are solid atmospheric particles emitted from organisms, including bacteria, fungi, protozoa, spores, algae, viruses, and microbial fragments. [52] These particles can be released to the atmosphere through both natural (e.g., resuspension by winds and bubble-bursting mechanisms on ocean surfaces) and anthropogenic activities (e.g., agricultural practices) [52]. Considering that Yangon is located in a coastal area, it was likely that those bioaerosols were emitted along with sea spray aerosols [53] and partially contributed OC to the ambient air.

#### 3.2.3. Combined Results from All Source Apportionment Methods

**Figure 9:**
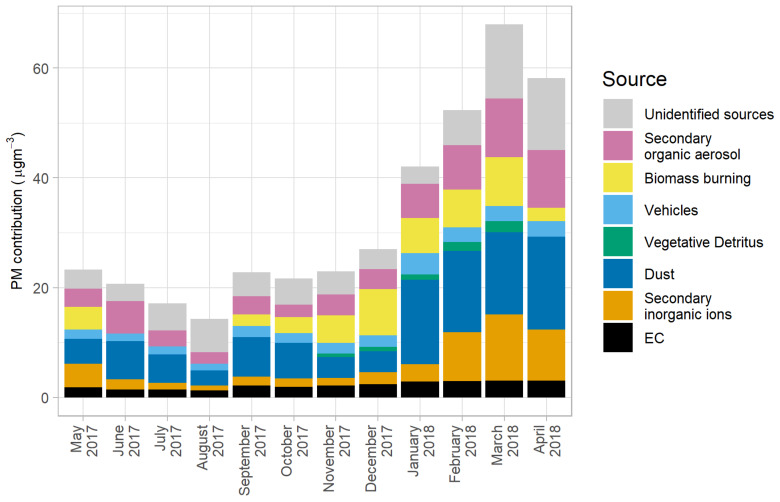
Monthly source contribution to PM_10_ mass estimated using the CMB model in Yangon, Myanmar.

Monthly source contributions to OC computed from the CMB model were converted to source contribution to PM_10_ mass using the specific OC/PM factor of each source profile. The contribution from the unidentified OC source was used to calculate SOA using an OC/PM factor of 2 [22]. EC, SIAs, and dust were also included as sources of PM_10_. Finally, the mass difference between the total gravimetric PM_10_ mass and the sum of all contributors was used to represent the unidentified sources, as seen in Figure 9.

The results from the CMB model and mass reconstruction reveal that dust was considered as the major primary source in every season. Dust contributions to PM_10_ were 9.5 ± 3.3 (26.2%), 12.2 ± 3.9 (24.4%), and 5.7 ± 0.8 (29.6%) µg/m^3^ in the winter, the summer, and the rainy season, respectively. In summer, SIA and SOA also became major sources contributing to PM_10_ mass with contributions of 8.5 ± 2.3 (17.1%) and 8.2 ± 2.4 µg/m^3^ (16.4%), respectively. In addition to dust, during the winter months biomass burning was the second largest source of PM_10_, contributing 6.7 ± 0.7 µg/m^3^ (18.6%), followed by a wintertime SOA contribution of 5.5 ± 1.1 µg/m^3^ (15.1%). The only time vehicles were considered to be a major source was during the rainy season, when they only contributed 1.6 ± 0.2 µg/m^3^ (8.1%), below dust and the second most important source, SOAs, which contributed 3.3 ± 0.7 µg/m^3^ (17.2%). These results illustrate the importance of quantitative source apportionment using chemical data for identifying major ambient PM sources. Yi et al. [2] conducted a preliminary study by using PM_2.5_ personal sensors along roads in Yangon townships and hypothesized that roadway emissions were the most important source, but with only ambient PM mass data, that hypothesis could not be corroborated.

## 4. Conclusions

To obtain a better understanding of the sources of coarse atmospheric particulate matter in Yangon, Myanmar, source apportionment using the Chemical Mass Balance (CMB) model was applied to chemical composition data collected from the one year of ambient PM_10_ samples, collected from May 2017 to April 2018. The results presented here suggest that PM_10_ concentrations in Yangon were affected by both meteorological factors (e.g., precipitation amount, storms, and temperature) and human activities (e.g., firewood burning and yard waste management). The highest PM_10_ mass concentration was observed in the summer, followed by the winter, potentially due to the strong winds and thunderstorms occurring during those times which resuspended the particles. The lowest mass concentration was observed in the rainy season when rain contributed to particle removal via scavenging. The main components identified in these PM_10_ samples were dust and organic matter (OM), which showed seasonal variations, as well as organic molecular markers such as levoglucosan and PAHs. A principal component analysis (PCA) using trace element data identified five potential sources of PM_10_: dust, secondary aerosols, metal manufacturing, biomass burning, and roadway emissions. Further investigation using source apportionment by the Chemical Mass Balance (CMB) model confirmed that the main sources of PM_10_ concentrations in Yangon were dust, secondary inorganic aerosols (SIA), and secondary organic aerosols (SOA). Additionally, biomass burning was an important source in the winter months. The findings presented here highlight the importance of a chemical composition study to inform policy decisions. By using source apportionment of particulate matter, policymakers gain information about which sources can be managed, which sources should be prioritized, and broadly, what should be done to reduce the adverse effects of atmospheric PM on human health and the environment.

## Figures and Tables

**Table 1:** Results of Principle Component Analysis (PCA).

Factor	Variance Explained (%)	Determining Species	Potential Sources
**1**	54.08	**Al**, **wi-K**, **Sc**, **Ti**, **Cr**, **Fe**, **Co**, **Sr**, **Y**, **Mo**, **Cs**,	Dust
		**Ba**, **La**, **Ce**, **Pr**, **Nd**, **Sm**, **Eu**, **Dy**, **Ho**, **Yb**, **Lu**,	
		**W**, **Pt**, **Th**, **U**, WIOC, Nitrate, Mn, Rb	
**2**	13.84	**Sulfate**, **Ammonium**, K, Mn, Se, Tl	Secondary aerosols
**3**	8.75	**Cu**, **Sn**, **Sb**, Ag	Metal manufacturing
**4**	8.54	EC, WSOC	Biomass burning
**5**	5.23	Pb, Zn	Roadway emission

wi = water-insoluble; **bold** = > 0.8 loadings.
